# An integrative review of social and occupational factors influencing health and wellbeing

**DOI:** 10.3389/fpsyg.2015.01281

**Published:** 2015-09-01

**Authors:** MaryBeth Gallagher, Orla T. Muldoon, Judith Pettigrew

**Affiliations:** ^1^Department of Clinical Therapies, University of Limerick, Limerick, Ireland; ^2^Centre for Social Issues Research, Department of Psychology, University of Limerick, Limerick, Ireland

**Keywords:** occupational science, occupational therapy, social structure, social identity, social class, wellbeing, activities of daily living

## Abstract

Therapeutic approaches to health and wellbeing have traditionally assumed that meaningful activity or occupation contributes to health and quality of life. Within social psychology, everyday activities and practices that fill our lives are believed to be shaped by structural and systemic factors and in turn these practices can form the basis of social identities. In occupational therapy these everyday activities are called occupations. Occupations can be understood as a contextually bound synthesis of meaningful doing, being, belonging and becoming that influence health and wellbeing. We contend that an integrative review of occupational therapy and social psychology literature will enhance our ability to understand the relationship between social structures, identity and dimensions of occupation by elucidating how they inform one another, and how taken together they augment our understanding of health and wellbeing This review incorporates theoretical and empirical works purposively sampled from databases within EBSCO including CINAHL, psychINFO, psychArticles, and Web of Science. Search terms included: occupation, therapy, social psychology, occupational science, health, wellbeing, identity, structures and combinations of these terms. In presenting this review, we argue that doing, being and belonging may act as an important link to widely acknowledged relationships between social factors and health and wellbeing, and that interventions targeting individual change may be problematic.

## Introduction

Occupational therapy, like psychology has tended to view one’s health and wellbeing as situated within the individual and largely determined by the internal motivation, abilities and constitution of that individual. Despite these disciplines espousing a biopsychosocial model and recognizing the influence of a range of factors on health, the individual often remains the focus of intervention and attention. Problematizing the individual obscures the potential for occupational therapy to address health and wellbeing concerns resulting from occupational inequities within communities and populations (Rudman and Dennhardt, 2008; Ikiugu and Pollard, 2015). Ergo, Occupational therapy scholarship and practice is deepening its understanding and consideration of the sociocultural context.

Social psychology has had a longstanding appreciation of the sociocultural context and particularly how social groups to which an individual belongs inform the individual’s identity, their behavior (Tajfel, 1981), and as such, their participation in occupation. Notwithstanding recognition of a range of contextual factors, individual behavior remains central to much scholarship and intervention within psychology and occupational therapy. A deeper analysis of how occupational therapy and social psychology study individuals, collectives, actions and structures within social contexts and how this influences health and wellbeing, will highlight the complementary nature of the two disciplines. It will also direct warranted, and much needed, attention to the social in the biopsychosocial perspective through collaboration.

## The Disciplines: Defined and Integrated

Here we present integrated disciplinary understandings of human action within a social world, arguing that interventions to improve health and wellbeing must consider that an individual’s identity and what an individual chooses to “do,” can neither be separated from each other, nor from the social structures that inform each of these entities. Doing is synonymous with occupation (Wilcock, 1999) the medium central to occupational science and to the discipline of occupational therapy. Occupational science is the study of humans as occupational beings (Yerxa, 1990). Introduced in 1989, it is the academic discipline that underpins occupational therapy. The profession of Occupational therapy was founded early in the 20th century, though its philosophical base emanates from a range of 18th and 19th century movements, including the mental hygiene and the moral treatment movements. These philosophies supported the idea that those living in asylums, who had been subjected to poor and inhumane treatment, were worthy of receiving care. Specifically, use of planned activities within a daily life regimen was introduced to imbue compassion through “normalcy” into the lives of those experiencing mental illness (Wilcock, 2006). Later in the 19th century, the arts and crafts movement, as a counter to industrialization, saw engaging patients in occupations, such as craftwork as moral and curative (Reed, 2015). Subsequent then to WW1, occupational therapists also became providers of rehabilitation services to injured or disabled veterans who faced a range of difficulties upon their return home. Combining these historical perspectives, occupational therapy today is about enabling engagement in occupations that promote health, wellbeing and participation in life for all people. To understand the significance of occupation in the lives of humans and the relationship of occupation to health and wellbeing, it is useful to describe occupation as a meaningful synthesis of doing, being and belonging (Wilcock, 1999; Rebeiro, 2001; Hammell, 2004). This will be discussed in detail later in the paper. Here it is important to know that it is through our occupations that we connect with the world and create meaning, enabling us to live well, healthily and ultimately to survive (Wilcock, 1999). Additionally, that this “doing” and its associated therapy, occupational therapy, is often concentrated on individualized interventions.

The focus of social psychology is on understanding how individuals think, act and feel when in social situations. Early social psychology was influenced by philosophical beliefs about human behavior as governed by rationality versus irrationality and social behavior based on utilitarian needs or innate social tendencies (Goethals, 2003). If human behavior was guided by abilities beyond one’s own mind, then the objective of social psychology was to apply a scientific method of inquiry to questions of social interaction and social influence. The objective of which was to establish principles of social behavior and by doing so, enable the creation of optimal societal conditions with maximum benefits to members (Gergen, 1973). Accordingly, contemporary social psychology and social identity theory in particular, points to the relevance of social structures to what we are enabled or dis-abled to “do.” Within the social identity approach concepts, social relations, shared identifications and belonging are linked to social groups which in turn are related to, how we live and how well we are (Haslam, 2004). Psychology and the social identity tradition have tended to orient to cognition as an important substrate for health and wellbeing. Thus an orientation to occupation offered by occupational therapy, allows a reorientation to the importance of activity and practice to health.

To support this shift in perspective we will first discuss health and how it can be viewed through social and systemic factors. We then explore how social factors shape our identity and our occupations. Next we consider how contextualized occupations influence health and wellbeing. We then conclude, arguing that interventions that attend to how identities and groups anchor and motivate people are those most likely to gain traction and embed in everyday activities and practices leading to improved health and wellbeing.

## Health: A Role for Occupational, Structural, and Group Influences?

Health is defined as a state of complete physical, mental and social wellbeing and a resource for everyday life. An individual or group must be able to identify and realize aspirations, satisfy needs, and change or cope with the environment (World Health Organization, 1986). Health and wellbeing are understood to be promoted through the ability to make purposeful and meaningful every day choices about what to do (Townsend and Wilcock, 2004). Occupation in which people engage has long been known to influence health and wellbeing (Creek and Hughes, 2008). Engagement in meaningful occupation, a concept central to occupational science, has been identified, not only as essential to maintain health, but as essential for survival (Wilcock, 1999). The occupations, for example, of food shopping, cooking, and house building are integral to our ability to maintain our health and wellbeing. Additionally, occupation provides structure and routine to our days, contributes to our dynamic sense of identity, and keeps us connected to others and to the world around us (Hasselkus, 2006). These additional aspects have also been shown to be essential for our health (Koome et al., 2012; Gupta and Sullivan, 2013).

Perhaps because of the predominant culture of individualism in the West both occupational scientists (those who study occupation as a human endeavor), and psychologists often neglect the role of macro-social or group influences in our analysis and our interventions (Dickie et al., 2006; Pollard et al., 2008; Kinsella, 2012). Occupational science, occupational therapy and psychology in clinical settings are disposed to concentrating attention at the individual level. In recent years, the emphasis within psychology and social sciences more generally, has been on genetic and neurobiological explanations of human behavior. This has led scholars to try to identify individual characteristics of those who tend toward criminality (Sampson and Wilson, 1995) or are disease prone for example (Friedman and Booth-Kewley, 1987). The contemporary Western zeitgeist influencing much scholarly thought has, and for the most part continues to emphasize and problematize individuals as the source of social ills (Sampson and Wilson, 1995). However, there is increasing criticism of this perspective. Those questioning this orientation (Dickie et al., 2006; Laliberte Rudman, 2013) suggest that it neglects the value of occupation (or doing) with communities and society and limits the potential to address the broader contexts that enable or constrain participation in occupation. There is much evidence to suggest that attending to collective experience, as in co-occupations (occupations done by two or more people) and community occupations, also contribute to meaningful engagement. These observations further our realization of occupation as socially situated (Fieldhouse, 2012; Mason and Conneeley, 2012; Ramugondo and Kronenberg, 2013). Here we propose an alternate understanding of the psychology of health and human behavior which at a minimum requires multiple levels of analysis.

Occupation happens in a cultural, temporal and ecological context (Hocking, 2000). This situatedness of occupation is important for understanding the meaning of occupation and subsequently, its impact on health and wellbeing. Recent critical perspectives, by occupational scientists, of the individualization of occupation have proposed broader social theories as a means of supporting the science of occupation. Specifically, Cutchin et al. (2008) building on the work of early 20th century educationalist, John Dewey, define occupation as a relational action through which context, along with habit and creativity are coordinated toward a particular outcome. In other words, you cannot separate the individual from their environment (Kuo, 2011). Literature in occupational science using this relational or transactional perspective highlights the capacity of social structures to inform engagement in occupation and consequently, health. In Canada, Rudman (2005) analyzed newspaper articles examining how structural and discursive transformations of retirement shaped what retirees viewed as ideal and possible occupations. She found that political discourses were instrumental in shaping what occupations retirees believed to be possible. Further, she argued that the neoliberal message heard by retirees is that they are individually responsible for their own aging and, by implication, their own health outcomes. Galvaan (2015) and Gallagher et al. (2015) explored everyday occupational choices of adolescents living in very disadvantaged neighborhoods in two different parts of the world (South Africa and Ireland, respectively). Both found that daily occupational choices that at risk young people perceive as accessible to them are pervasively informed by the socio political, sociocultural and socioeconomic contexts in which they live. This perception by the young people predicates particular patterns of occupational engagement that may reinforce negative health behaviors.

Thus it can be said that we are highly sensitive to our social context and in particular to disadvantage or inequality and there is a growing evidence that this impacts health (Marmot, 2004). Measures of income inequality allow comparisons of the effects of inequality across nations. These measures include the GINI index, a commonly used measure of income distribution inequality, as well as indicators of the proportion of wealth owned by the richest proportion of the population, a key measure being the 20/20 ratio. The GINI index is based on income distribution of a nation’s residents and varies between 0 and 1 (with 0 indicating perfect equality of income distribution and 1 complete inequality). The GINI coefficient has been linked convincingly in a large number of diverse countries, to self-rated health (Mansyur et al., 2008) and morbidity (Kondo et al., 2009) for example. Consistently, research confirms that irrespective of how affluent or poor a nation is it is the level of inequality in a society that determines the steepness of the social class gradient in health (Marmot, 2004). As a consequence, wealthier nations are not necessarily healthier. Health depends on the level of inequality (Wilkinson and Pickett, 2009). Alternatively stated, in societies where the distribution of wealth is more equal, all social classes are healthier.

Marmot’s findings on the impact of inequality have important implications. While material disadvantage has consequence for our health, it is relative inequality that has the greatest impact on health. Our relative economic position vis-a-vis other people is interpreted and imbued with social meaning. Feeling valued, autonomous and appreciated in society and having access to social support are integral to relative social positions and these are all important factors in health and wellbeing outcomes. Furthermore these differences are not only apparent across nations; they are also evident across the social class gradient within nations. The very poor suffer most from inequality: life expectancy gets shorter and most diseases become more prevalent with each descending rung of the social ladder (Wilkinson and Marmot, 2003). In a longitudinal study of civil servants in 20 London government departments, the risk of anxiety and depression was evident in the social class gradient amongst both women and men. Both genders in the lowest employment grades had a significantly higher risk of poorer psychological outcomes than those in higher grades and the risk significantly decreased as employment grade increased (Wilkinson and Marmot, 2003). Thus it can be said that health is linked to not only structural conditions and material disadvantage but also one’s interpretation of this disadvantage and the subjective meaning with which it is imbued. This portends to a social psychology of health, where context and its associated meaning impacts on health and wellbeing.

## A Social Identity Framework: Collective Context, Identity, and Occupation

Social identity theory has been a hugely influential theory in the development of European social psychology. Identity can be understood as the active and dynamic understanding of self that people derive from interactions between themselves and their environments (Simon, 2004). Tajfel’s (1981) original formulation argued that social categorization or the tendency to label ourselves and others according to group characteristics is a pervasive social process. Often these labels are short hand methods of saying more about the group members than mere membership implies. Effectively the categorization allows a degree of stereotyping. Importantly this process of categorization is linked to extant social relations, categorization being most likely to occur spontaneously when social and political structures serve to embed group differences. Social identity theory is thus concerned both with the psychological and sociological aspects of group behavior.

Subsequent to categorization, of the self and others as group members, identification with the group develops. This process of identification means that individuals develop a degree of buy in into the groups to which they belong. This willingness to belong has emotional, behavioral and cognitive elements. Tajfel argued that those who identified more strongly with their group, felt better about the groups to which they belonged and as a consequence about themselves. More recently, researchers in this area have argued that the concerns that identity motives may be far more wide ranging and may include strategic and political concerns as well as processes that serve to bolster and support mental and physical health.

In this way identities can also be powerful psychological resources to deal with adverse social, educational and economic challenges (Muldoon and Lowe, 2012; Muldoon, 2013; Cruwys et al., 2014; Jetten et al., 2014). In a landmark paper, Haslam et al. (2009) identified the investigation of relationships between social identities, health and wellbeing as an important applied field of inquiry for contemporary social psychology. The idea of “social cure” was further developed by Jetten et al. (2012) who advocated the social identity approach as a perspective particularly well suited to application in the study of associations between social relationships, group memberships, health and wellbeing.

Not all social identities are the same, however. So although much of the early work relating to the nature of groups and their consequences employed a minimal group paradigm, increasingly psychologists have paid attention to real world social groups. The application of the social identity model to such real world groups is of course the truest test of the model. Over the past decade, research has particularly attempted to recover the “lost nation of psychology” for example by examining the specificities of national identity (Reicher et al., 1997). These attempts have been informed by the work of Billig (1995) on the “banality” of nationalism. Billig (1995) identified banal aspects of social identities. Identities are often unexpressed and unrecognized but nevertheless present and available for mobilization if and when required. These banal or background identities hinge on feelings of belongingness. They are the groups to which we affiliate; the groups to which we feel we belong that are understood as making us who we are. Family and nation are perhaps two obvious examples. These identities have been referred to as affiliative identities (Stevenson and Muldoon, 2010).

Another distinction in types of social identities, drawn by Deaux et al. (1995) is between identities founded upon relationships and identities associated with occupation. This observation has also been made in the literature in other disciplines where the self is understood as a conduit through which “who we are” is constructed via “the social groups we are immersed in” (Lieberman, 2013, p. 191). This second type of social identity considered here has been described by Houser-Marko and Sheldon (2006) as “the self-as-doer” construct. These identities are actively constructed in everyday ways and are actively claimed (Stevenson and Muldoon, 2010). Ashmore et al. (2004) distinguish between ascribed identities, such as gender, and achieved identities, such as occupation. Ascribed identities are similar to the types of affiliative identities detailed previously whereas achieved identities include those “self-as-doer” type identities identified by Houser-Marko and Sheldon (2006). Occupation can be seen as a fundamental component of achieved identities as what we do informs our identity and likewise identity influences occupation, not just what we do, but how we do over time (Christiansen, 2000). These constructive components of identity are more likely to be strategically deployed in the manner suggested by self-categorization theory (Reicher and Hopkins, 2000). These identities are projects ongoing in the sphere of conscious awareness and day-to-day discourse. This strategic deployment of actions in the face of occupational challenge is referred to as occupational adaptation (Schultz and Schkade, 1997). The process of occupational adaptation in which one’s identity and wellbeing are central, has been posited not only as a reaction to a disruption or a transition, but as a conscious everyday strategy to advance one’s occupational choices, thus reinforcing self-concept and supporting wellbeing (Nayar and Stanley, 2015). Occupation is the nexus of these self-as-doer identities enabling positive occupational identity construction and enhancing occupational competence (Kielhofner, 2002, p. 121). So from several theoretical points of view, ranging from the social constructionist (e.g., Butler, 2003) to the neuropsychological (Wilson et al., 2009), to the social identity approach (Klein et al., 2007), to occupational therapy (Wilcock, 1998; Hammell, 2004), identity is presented as intimately intertwined with “doing.” Some identities require performance, in order to be sustainable. Identities must be capable of expression and generally require recognition by others in order to be viable (Klein et al., 2007).

Taken together, a contemporary social identity approach highlights the important relationship between extant social structures and groups and the social identities we subsequently develop. It is increasingly becoming apparent in a growing literature that these identities are linked to health and wellbeing. However, identities are not homogenous. The available literature would appear to suggest that identities that support “belonging” and those that support “doing” may both impact on health although this impact may differ. Interestingly this distinction between doing and belonging is one that has resonance in the discipline of occupational science and it is to this research we now turn.

## Occupational Science: Meaningful Being Doing, and Belonging in Context

Central dimensions informing therapies are the sense of “doing,” “being,” and “belonging” and the inherent meaning of each for the individual. The founding of occupational science has contributed much to our empirical understanding of occupation and how it is experienced by people. The academic notion of occupation as dimensions of meaning rather than a therapeutic taxonomy of self-care, productivity and leisure creates opportunity for nuanced distinctions between doing, being and belonging (Hammell, 2004) that has further enhanced our understanding of the complexity of occupation.

### Being

Before we consider doing and belonging, we will briefly address “being” as understood in occupational science. As a meaningful dimension of occupation, being can be considered a focus of attention from active to passive doing; a dimension of occupation that creates a space for being true to ourselves, our identity, and appreciating what is unique about us within our interactions with others (Wilcock, 2006). For example, the moment during the morning school run when you stop focusing on the clock, the traffic and getting to work on time and appreciate the journey as time spent with your children, being a parent. It is seen as introspective and reflective and is often over looked in our “doing” filled lives (Hammell, 2004). The current popularity of mindfulness practices to maintain mental health and improve depression support the idea of being in the moment and suggests that we spend too much time thinking about what we did in the past or what we have to do in the future (Kabat-Zinn, 1994). Mindfulness then can be a mechanism for connecting what we do with who we are; our “doing” with our “being.” In today’s busy and technologically imbued context, the balance between doing and being can easily be lost. In this situation, people can be enabled to find occupations that create time and space for reflection and meaning making. Mindfulness and flow (Kabat-Zinn, 1994; Csikszentmihalyi, 2014) are concepts that advocate a presence in and awareness of occupation in the moment, and have been suggested to influence wellbeing (Reid, 2011). These opportunities for being can restore occupational balance and the natural equilibrium of our biological, psychological and occupational selves (Wilcock, 2006). Conversely, there are those deprived of “doing” for whom “being” is too consuming and central as individual’s lives may lack opportunity or resources for meaningful engagement in occupation, such as refugees and asylum seekers (Whiteford, 2000) those in secure forensic settings (Farnworth et al., 2004) or those living in poverty. There is a dimension of “being” that goes beyond introspection and relates to how we are situated in the world. In considering this aspect of being, the social identity framework just discussed offers insight into how our individual selves are situated within structures and often informed by the labels promoted by these social structures. For example “being” a mechanic, and “being” a doctor each portray different identities that when interpreted within the social world may impact how others view our worth and inform our own sense of self.

### Doing

Doing is so central to human existence that life cannot possibly be imagined without it (Wilcock, 1999). Meaningful “doing” is therapeutically used to engage with people when disruption in their lives impacts on their ability to do every day things. For example, when circumstances of forced migration and consequent resettlement adversely disrupt familiar and safe roles and routines, occupations, such as, finding the grocery store, cooking culturally cherished dishes and transferring existing capabilities into a new context can facilitate the re-development of life skills that enable this transition; particularly when the reconstruction of everyday meaning and the enculturation of newly emerging identities are necessitated by the broader resettlement context (Suleman and Whiteford, 2013). Likewise broader considerations of meaningful “doing” are apparent when participating in occupations at the community level. Community gardening has been shown to minimize social exclusion through the gardening group’s ability to establish and maintain links with the wider community and by individuals developing knowledge and skills (e.g., horticulture) that are relevant to a wider social circle (York and Wiseman, 2012). As well as challenging negative stereotypes by developing roles that lend to positive identity formation (Mason and Conneeley, 2012). Further, social farming, in which mental health service users supported socially and practically by the farmer, participate in animal assisted interventions on a small farm has been shown to improve a range of symptoms associated with poor mental health (Berget et al., 2011). This social inclusion initiative has been shown to lessen anxiety and depression and increase self-esteem, self-efficacy and coping (Berget and Braastad, 2011).

Fundamentally, what we do influences our health. From the broader social levels just described to the very basic level when we do things like eat well, sleep well, attend to hygiene and avoid high risk activities, like driving without a seatbelt, cycling without a helmet or smoking we can contribute to maintaining our health and prevent ourselves from accidents or ill health. Indeed, the world’s top causes of death, such as heart disease, lung disease and stroke are largely modifiable and preventable through engagement in health promoting occupations, such as healthy eating routines and participation in moderate physical activity (World Health Organization, 2014). However, as previously stated occupations are complex and embedded in context. For example, it is not necessarily easy for people on low incomes to eat well. Eating well can be expensive, as healthful diet routines are often more costly (Rao et al., 2013). Likewise, it is not easy for someone who is homeless to sleep well considering the challenge of finding a safe, warm, and dry place to sleep (Chang et al., 2015). People must have the resources and the capabilities to “do” and “belong” in ways that enable them to look after their own health.

### Belonging

Ultimately, humans are social beings and as such are embedded in collectives to which belonging is central for health and wellbeing. “Belonging” is about feeling safe, and worthy of acceptance and love (Rebeiro, 2001). Belonging as a dimension of occupation arose from Rebeiro’s (2001) study of the meaning of occupational engagement for a group of women with mental ill-health. Rebeiro found that belonging needs were critical to life satisfaction, as it was only when the women in her study felt supported and safe in their environment that they were able to experience meaningful engagement and participation. Wilcock (2007) acknowledged belonging as a dimension of occupation because of the connectedness people experience to one another when engaged in occupations together and the subsequent impact of these relationships on health. The need to belong takes precedence in informing daily occupational choices made by adolescents living in socioeconomically disadvantaged urban areas (Gallagher et al., 2015). Indeed, the sense of belonging experienced through social occupations is so powerful that Glass et al. (1999) longitudinal study of older people found it increased life expectancy. Conversely, belonging needs have also been identified as potentially stigmatizing. Hvalsøe and Josephsson (2003) found that disabled individuals who needed to be with others for assistance due to the challenges of doing things on their own, experienced feelings of differentness and therefore potentially not belonging. Given the importance of belonging to health and wellbeing, foregrounding the individualization of occupation and backgrounding the collective, social, economic and political structures may act to advance health inequities (Laliberte Rudman, 2013). Occupational science is at its genesis in understanding an individual’s occupational self as inextricably and relationally embedded within their context (Dickie et al., 2006). This makes social identity decidedly relevant to occupational science.

### Meaning

If we add to these dimensions the meaning that individuals derive from their group memberships, as well as from occupations, we gather another perspective of the relationship between belonging and health and wellbeing. Van Nes et al. (2012) studied meaningful co-occupations for older couples as told through photo-stories and found that both being and belonging were central to enjoyment of the co-occupation. When viewing an image of themselves with arms linked on one of their walks—one half of the couple interpreted this as the closeness they experience and the sameness of their steps, while the other partner interpreted this as a way of keeping his balance. These interpretations of the walk represented both the shared and personalized meanings of the occupation. For both it was about doing something meaningful together, contributing to their identity as a couple, despite the different explications. The meaning of occupation is significant as many disciplines have identified meaning as essential to quality of life (Eklund et al., 2012). Even when people do not view their occupations positively or indeed when they are subjected to horrible occupational experiences, the search to make meaning of it can lead to survival (Frankl, 1985). The meaning of the occupation to the individual is critical to the occupation’s potential as an agent of change (Wilcock, 2006). Using meaningful occupation therapeutically enables the skills and abilities necessary for participation in life. For example, Merryman et al. (2012) study of an occupation centered summer camp for at-risk youth demonstrated that occupation based programming, within an enriched environment that enabled campers’ ability to make choices, led to improvements in skill competence (social skills, thinking skills, personal values and positive self-identity), the capability to make positive occupational choices, and increased resilience. These improvements were effectively sustained upon return to the high-risk environments from which the young people came. Improvements in perception of choice and self-identity may be instrumental in envisioning alternate ways of being that lead to better social, economic and health and wellbeing outcomes.

Occupational science has also led to greater attention to the broader contexts that inform and shape occupational engagement. As a result we are learning that individualized interventions targeting impairments enabling people to “do” may be misdirected. For example, research with wheelchair users consistently highlights societal barriers as equally, if not more disabling, to participation than physical barriers (Ripat and Woodgate, 2012). Political barriers to occupation have also been highlighted. For example, policy decisions regarding support for single mothers whereby participation in any paid employment means mothers risk losing benefits that enable them to provide for their children. These decisions keep single mothers from engaging in occupations that could potentially increase their self-esteem and develop their identity as a contributor to society, thus increasing their sense of belonging. As we develop our understanding of the ways in which the socio economic, socio cultural and political contexts both constrain and facilitate people’s ability to participate in occupations of meaning, the more interventions can address the actual barriers to participation that negatively impact on health and wellbeing.

## A Particular Case: Social Class, Occupation, and Identity

While the degree to which economic or social mobility can be achieved is debatable, socio-economic identities are more likely to be seen as malleable and therefore must be actively claimed (Stevenson and Muldoon, 2010). Social identification is believed to be underpinned by social comparison and distinctiveness processes. Given the more malleable nature of social class position and the potential for misunderstanding of relative positions, achieving comparison and distinctiveness via doing, being and belonging is essential to maintaining social class positions. In this way, occupation can be seen as central to definitions and understandings of social class and social class can be seen as instrumental in informing occupation. Occupational identity has both individualistic and conformist components (Rudman and Dennhardt, 2008). Conformist occupational identity aligns strongly with affiliative social identity and is evident when occupational choices reflect the expectations of the sociocultural environment. For example, when gang members meet group expectations by choosing to embody similar characteristics, such as dressing in similar clothing or sporting the same tattoo. In essence, claiming the gang member identity. Individual occupational identity is exhibited when occupational choices are not in keeping with the sociocultural context (Rudman and Dennhardt, 2008), thus exhibiting distinctiveness. The presentation of social class as achieved, earned or even chosen, is often linked to the development of negative social identities, or stigmatized identities particularly for low status groups (Fiske et al., 2002). Despite widely held categorization, identification and stereotyping of income groups (Brattbakk and Hansen, 2004; Warr, 2005; McCulloch et al., 2006; Nayak, 2006), the dominant presentation of social class identities as individually achieved inhibits our understanding of this stereotyping as a group based phenomena. Much like understanding occupational identities as ascribed to individuals inhibits our understanding of structures informing an individual’s occupations. These phenomena are socially situated and therefore can be shaped by context in the way that contemporary discourses in social media can promote particular subjectivities and alienate others, and thus set standards for ways of “being” or identities (Rudman, 2005).

So while identities can be important resources for health and wellbeing, stigmatized identities are often associated with economically disadvantaged groups. Labeled with negative identity attributes such as helplessness or anti-sociality which are then used to justify and perpetuate social exclusion (McNamara et al., 2011). Coping with stigma generally involves trade-offs (Major and O’Brien, 2005). Typically the perception of discrimination is seen to consolidate a shared identity that groups can then use as a collective resource- a form of psychological empowerment (Haslam and Reicher, 2006). Indeed being a member of a range of social groups is a key aspect of the development of “social capital”: The network of alliances and relationships within a community or organization that contribute to its reputation, membership involvement, loyalty and commitment (Haslam et al., 2003). Feeling positive bonds of identity with others within a group is the basis for extending and taking help from group members and underpins community involvement and social responsibility (Levine et al., 2005; Reicher et al., 2007). However, members of disadvantaged communities are members of fewer social groups, have weaker bonds to these groups and a smaller range of opportunities to engage with and contribute to community life and have less influential connections. Such diminished social capital and marginalization from meaningful occupation, negatively impacts health and wellbeing (Townsend, 2012). Individuals lack opportunities to develop their potential, to foster their social inclusion and as such, become susceptible to low mood, low self esteem, isolation, and limited perception of possible futures.

In situations like this, when emotional and psychological resources for coping are depleted; occupation can be a conduit to meaning via our relationship with ourselves and others (Eakman, 2015). Occupation centered interventions can lessen psychological distress (Kohn et al., 2012), improve mood (Graff et al., 2007) and enable individuals to facilitate the orchestration of daily occupations that promote wellbeing (Doble and Santha, 2008). Attributes such as self-concept, self-esteem, and motivation as precursors to coping are developed and mediated through participating in meaningful daily occupations (Brown and Stoffel, 2015). Through “doing,” individuals find meaning in their lives, interact effectively with others, develop skills for making decisions, clarify values and beliefs, cope with stress and adapt to changes in life circumstances and demands (Wilcock, 2006). By developing a range of skills through occupation, self-esteem and mastery can be developed, potentially lessening the need for trade-offs in coping with stigma. Human engagement in occupations is a complex phenomenon based on individual and cultural meanings and therefore has the potential to counter stigmatized identities and their potential to negatively impact health outcomes. For example, male carers of a preschool child, along with their child, participated in a gardening project in a disadvantaged community. The findings revealed that fathers created stronger bonds with their child, promoting their father identity. Additionally, they developed social bonds with other fathers, enhancing their social identity as involved fathers, thus, minimizing the stigma and stereotypes associated with those from a disadvantaged background (Mason and Conneeley, 2012). In this way, occupation can be seen to have important consequences for health and wellbeing via “doing,” “being,” and “belonging” as shaped by its situatedness in people’s lives.

## “Becoming” as Means to Health and Wellbeing

Complementary to aspects of “doing” “being” and “belonging,” “becoming” implies a sense of movement toward a future way of being. In this dimension of occupation, the capacity to create new identity narratives and to move toward becoming all who one needs and wants to be is enabled (Hammell, 2004). In this paper we have reviewed and aligned literature from social psychology, occupational therapy and occupational science to support the argument that what we do is inextricably linked to who we are. Further that, who we are is inseparable from the sociocultural context in which we exist and that this combination is linked to health and wellbeing. In other words, we “wear our environment like a glove,” (Rowles, 2000, p. 52S) and our doing, being, belonging and becoming are spread across the social networks we inhabit. These ideas are not new, however, we believe that our synthesis of these interrelating perspectives can inform thinking and interventions, in both the clinic and the community as we come to more fully recognize the predominance of social identity and structures in knowing individuals authentically and engaging them in occupations to improve their health and wellbeing.

Two classic examples of interventions from the respective disciplines are presented here to illustrate an integrative acumen proposed in the preceding sections. The first example relates to a typical occupational therapy intervention in the home of an older person, whom we will call Maureen, who has experienced a fall. The second example is a classic psychology intervention for Diane, a young mother of three children, who is experiencing significant stress and anxiety.

### Occupational Therapy After a Fall

The standard offering from the occupational therapist in this situation is to advise Maureen to remove the small throw or scatter rugs from throughout her home to make it safer and lessen her likelihood of a fall. While the rationale for this practical act makes sense to the worldview of the therapist, it does not genuinely consider the reality of life for, and the situatedness of, this particular older person. Maureen happens to be poor and so cannot afford to keep her heating on to ensure her home remains consistently warm and comfortable. The rugs provide both an actual warm covering of the cold floor on which Maureen must walk and also provide a warm “coziness” to the atmosphere that Maureen finds comforting. Additionally because Maureen is poor, she cannot afford to carpet the whole floor, and these small rugs have done the required job for years. Finally, these throw rugs are the manner of demonstration for Maureen’s pride in her home and her identity as a homemaker, as she has carefully chosen each rug for a specific and personally meaningful reason. Given her low socioeconomic position, this threat to her homemaker status is significant and given that the therapist is most likely middle class, there becomes an even greater need for cultural sensitivity and awareness. In reconsidering the intervention based on a situated or contextualized understanding of who Maureen is, the therapist may wonder whether this intervention is likely to be successful at all. Certainly in making this suggestion, there should be an appreciation of the threat of the intervention to Maureen’s occupational and social identity, and warrants a broader discussion of how to make Maureen safer in her home.

### Psychology Intervention for Significant Stress and Anxiety

There is a belief when working with a person experiencing significant stress, that the person has a need to “be in the moment” and often this is the advice prescribed. Again, a very sound and well evidenced piece of mindfulness advice. However, for Diane, a young woman with three small children and an unreliable partner who does not contribute to living costs, this intervention may do little to alleviate the intensity and severity of Diane’s stress. Diane’s primary occupational identity is as a mother and a provider to her children. Diane works incredibly hard to ensure that her children’s need for good nutrition is met and this process consumes much of her time and a lot of her income. When one of her children is crying that his shoes are too small and his foot is painful, Diane needs to manage how to finance this unexpected cost on top of all her other usual expenses. When in this acutely stressed state, she is told to “be in the moment”; this may become another perceived responsibility. All of her thoughts and energy relate to getting those new shoes, making sure her child is not in pain and being a “good” mother. The notion of putting those plans on hold to be in the moment may be more than Diane can cope with. Again, there is a likelihood that class differences mean the therapist has no experience of the stress that comes with having no money and never having to consider the material realities of life in the same way as Diane. This intervention requires reconsideration based on the understanding of what Diane needs to “do” to maintain her most important identities of mother and provider. Even having a greater understanding of and compassion for exactly what is being asked of Diane to “be in the moment” is likely to make this intervention more effective and potentially more sustainable.

These simplistic examples are used to highlight the importance of informed decision making for interventions that incorporate understanding of “being in place” and knowing someone authentically. Such interventions will be more effective as they acknowledge the transactional, relational, dynamic messiness that is the human experience. Interventions and approaches must move beyond linear and reductionist tactics that situate the problem within the individual. They should address the person as they exist in a sociocultural world, the “real world.” In this way, they will be more person centered, more in keeping to the lived experience and as such, much more likely to be sustained as a means of promoting health and wellbeing.

Considering the importance of social factors, and in particular social identity, on our occupations and health and wellbeing, our interventions require mobilization beyond individualized conceptions of health, appreciating that a person is never separate from their context. Interventions are not about the individual, they are about the individual’s transactional relationship with the group, and the group’s place in the context. You cannot enable one’s “becoming” without recognition of the social factors both inside and outside the group. A good example of this is the Brazilian practice of “social occupational therapy” (Galheigo, 2005), which emphasizes collective action, and integration between individuals, groups, communities and society. This way of working in Brazil developed in response to both growing social inequality and an acceptance of social responsibility and social transformation as part of occupational therapy practice in Brazil in the 1970’s. In developing this practice, therapists recognized that concepts such as individual normality and functionality from rehabilitation would not work in the social context. They needed to acquire “tools to interpret the connections between personal and social realities” to enable a more innovative practice. In this way the therapists learned to enable participation in “counter hegemonic activities” through which individuals might be facilitated to take alternative paths from “appalling life conditions” (Dias Barros et al., 2011, p. 209). These conditions implore interdisciplinary perspectives to address social constructs such as marginalization, disadvantage and disaffiliation. Constructs in which social identity and occupation are central and where the potential for both oppression and liberation exist.

## Conclusion

In summary, we have proposed a multi -level and integrative model of understanding that who we are and what we “do” are situated in a social world. Further, we have demonstrated that this social situatedness informs our identity and our occupations in ways that directly influence our health and wellbeing. It is our assertion that occupation drives identity processes in and of itself, but also because of the relationship between doing or practice and our sense of ourselves and our relationships to others. Meaningful occupation provides the basis for social participation (Wilson et al., 2009). Occupation then carries with it the potential for considerable therapeutic utility providing as it does a basis for identity (re)construction (Clark, 1993; Walsh et al., 2015). Attention to the ways in which occupation is situated and embedded within structures and context is critical to any perspective on health (Wilcock, 2006). It is through understanding this relationship that we can create contexts for enabling individuals, communities and society to engage in meaningful occupations that promote participation, health and wellbeing. The complexity of occupation, its transactional nature, and its situatedness opens a door to enhancing our understanding of being well and staying well in social world.

Everyday social lives are complicated. While we are all individuals and sometimes act as individuals, we are also group members and sometimes our behaviors or occupations are enabled or disabled by these (racial, ethnic, religious, gendered, or socio-economic group) memberships. Central to the understanding of human behavior is the consideration of whether individuals are acting as individuals, or as a consequence of their group memberships. A failure to attend to group level influences in determining occupations misses a key level of analysis. This level of analysis though obvious to many social scientists is frequently bypassed in clinical practice. This can result in a failure to focus on wider structural level influences that drive behavior through valued social identities. This additional level of analysis is an important potential avenue through which health promoting interventions and everyday practices can gain traction. This analysis therefore links macro-social or structural level factors with health and wellbeing outcomes drawing from occupational perspectives and social psychology. In short, our memberships of social groups, be they national, racial, ethnic or socio-economic, are central to understanding everyday behavior, or occupation, and that these groups and these behaviors have consequences for both physical and psychological wellbeing.

### Conflict of Interest Statement

The authors declare that the research was conducted in the absence of any commercial or financial relationships that could be construed as a potential conflict of interest.
